# MMP‐8 in Peri‐Implantitis: A Cross‐Sectional Study on Genetic Polymorphisms and Enzymatic Activation

**DOI:** 10.1111/jre.70026

**Published:** 2025-08-07

**Authors:** Ioannis Fragkioudakis, Christine Kottaridi, Michalis Paraskeva, Dimitra Sakellari

**Affiliations:** ^1^ Department of Periodontology and Implant Biology School of Dentistry, Aristotle University of Thessaloniki Thessaloniki Greece; ^2^ Department of Genetics, Development, and Molecular Biology School of Biology, Aristotle University of Thessaloniki Thessaloniki Greece

**Keywords:** aMMP‐8, gene expression, MMP‐8, peri‐implantitis, single nucleotide polymorphism

## Abstract

This graphical abstract illustrates a molecular cascade linking MMP‐8 gene polymorphism (−799C/T), upregulated mRNA expression in peri‐implant crevicular fluid, and elevated active MMP‐8 protein levels. These factors were independently associated with peri‐implantitis and may support molecular‐based diagnostics.

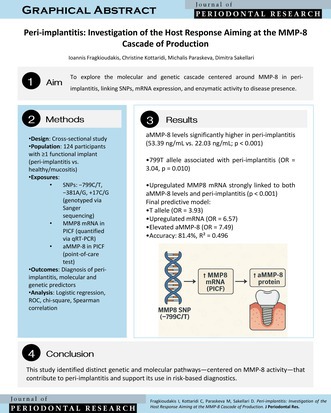

## 1 Introduction

The genetic and transcriptional factors regulating the elevated production of active‐matrix metalloproteinase‐8 (a‐MMP‐8) in peri‐implantitis remain insufficiently elucidated [1, 2]. Given the clinical relevance of MMP‐8 as both a biomarker and effector molecule in peri‐implant inflammation, this study aimed to explore a potential multilayered host‐response model involving gene polymorphisms, mRNA expression, and protein‐level activation of MMP‐8.

## 2 Methods

The present cross‐sectional study (ClinicalTrials.gov ID: NCT05711407) included 124 individuals with functional dental implants that had been loaded for more than a year. Participants were classified into peri‐implantitis and health/mucositis groups using current diagnostic criteria [3]. Peri‐implantitis was defined by the presence of bleeding and/or suppuration on probing, increased probing depth compared to previous examinations (if available), and radiographic bone loss ≥ 3 mm apical to the most coronal portion of the intraosseous part of the implant. Peri‐implant mucositis was defined as bleeding on probing without additional bone loss beyond initial remodeling. Clinical health required the absence of bleeding on probing and no progressive bone loss. Radiographs were used solely for classification purposes, with bone loss evaluated visually from the implant platform to the first bone‐to‐implant contact; no manual or software‐based measurements were performed. Diagnosis was based on full‐mouth and implant‐specific clinical examination, including six‐point probing per implant. One implant per subject was randomly selected to ensure unbiased site selection. Ethical approval was granted by Aristotle University of Thessaloniki (No. 115/25–05‐21), and all participants provided written informed consent.

Clinical parameters recorded included probing depth (PD), clinical attachment level (CAL), and bleeding on probing (BoP). Standardized periapical radiographs were taken using the long‐cone paralleling technique, and bone loss was visually assessed from the implant platform to the first bone‐to‐implant contact (fBIC).

Active MMP‐8 (aMMP‐8) levels in peri‐implant crevicular fluid (PICF) were measured using a chairside lateral‐flow immunoassay (ImplantSafe/ORALyzer, Dentognostics GmbH). MMP‐8 mRNA expression was analyzed via quantitative real‐time PCR (qRT‐PCR), normalized to β‐actin, and calculated using the ΔΔCt method. A fold change > 2.0 in MMP‐8 mRNA expression was considered indicative of upregulation for binary classification. This threshold was chosen based on commonly accepted biological relevance in gene expression studies, where a twofold change is often regarded as the minimal level of change likely to reflect true biological significance rather than technical or inter‐individual variability [4].

Genotyping of three MMP‐8 SNPs (−799C/T, −381A/G, +17C/G) was performed by Sanger sequencing using DNA from unstimulated saliva. Full sampling and laboratory protocols are detailed in the Data S1.

Statistical analysis was conducted with SPSS v27.0 (IBM, NY, USA). Categorical variables were compared using chi‐square tests, and continuous data were analyzed using the Mann–Whitney *U* test. A multivariable binary logistic regression model was used to identify predictors of peri‐implantitis, including: (1) −799 T allele presence, (2) MMP‐8 mRNA upregulation, and (3) elevated aMMP‐8 (> 20 ng/mL), along with smoking status and implant time in function. For regression analysis, a cut‐off value of 20 ng/mL was used to dichotomize aMMP‐8 levels, based on previously validated point‐of‐care studies demonstrating this threshold's diagnostic utility in identifying peri‐implant inflammatory conditions, including both mucositis and peri‐implantitis [1, 2]. This threshold does not distinguish peri‐implantitis specifically but reflects elevated host‐derived proteolytic activity indicative of active inflammation. All predictors were entered simultaneously (ENTER method). Model fit was evaluated using the Hosmer–Lemeshow test and Nagelkerke *R* ^2^.

Multicollinearity was assessed using the variance inflation factor (VIF); all VIFs were < 1.02 and tolerance > 0.98, indicating no collinearity.

## 3 Results

### 3.1 Participant Characteristics

Among 124 participants, no significant differences were found between the peri‐implantitis and health/mucositis groups regarding age, sex, or smoking status. However, peri‐implantitis cases demonstrated significantly deeper probing depths (PD), greater clinical attachment level (CAL) loss, higher bleeding on probing (BoP) percentages, and higher levels of a‐MMP‐8 compared to controls (*p* < 0.001; Table 1).

**TABLE 1:** Participant demographics and clinical parameters.

Parameter	Health/mucositis	Peri‐implantitis	*p*
Age (years, mean ± SD)	56.2 ± 8.3	57.9 ± 7.5	0.218
Smoking status (% current smokers)	24.1%	34.8%	0.128
Probing depth (PD, mm, mean ± SD)	2.94 ± 0.78	4.59 ± 1.22	< 0.001**
Clinical attachment level (CAL, mm, mean ± SD)	3.05 ± 0.81	5.21 ± 1.72	< 0.001**
Bleeding on probing (BoP, %, mean ± SD)	18.79 ± 24.17	57.58 ± 31.73	< 0.001**
Active MMP‐8 (aMMP‐8, ng/mL, mean ± SD)	22.03 ± 32.87	53.39 ± 49.70	< 0.001**

*Note:* Mann–Whitney *U* test for continuous variables; Chi‐square test for categorical variables.

** *p*‐values indicate statistical significance (*p* < 0.05).

Genetic variation in *MMP‐8* was assessed as an exploratory, hypothesis‐generating analysis. Among the three single‐nucleotide polymorphisms (SNPs) examined (−799C/T, −381A/G, +17C/G), only −799C/T displayed allelic variability in the study cohort; the remaining SNPs were monomorphic. Given the limited variation and exploratory scope, no correction for multiple comparisons was applied.

The −799 T allele was significantly associated with peri‐implantitis (OR = 3.04; 95% CI: 1.31–7.10; *p* = 0.010), although it was not related to MMP‐8 mRNA expression levels (*p* = 0.357). MMP‐8 mRNA was upregulated in 71.2% of peri‐implantitis cases compared to 12.5% of controls, indicating a strong association with both elevated aMMP‐8 levels and a peri‐implantitis diagnosis (*p* < 0.001 for both). The presence of the T allele was not significantly associated with MMP8 expression (OR = 1.50, *p* = 0.358). However, MMP8 expression was significantly associated with increased protein levels in PICF (OR = 4.44, *p* = 0.002).

Multivariable binary logistic regression identified three independent molecular predictors of peri‐implantitis: presence of the −799 T allele (OR = 3.93; *p* = 0.020), upregulated MMP‐8 mRNA expression (OR = 6.57; *p* = 0.001), and elevated aMMP‐8 levels (> 20 ng/mL) (OR = 7.49; *p* < 0.001). This model demonstrated strong discriminatory performance, with an overall classification accuracy of 81.4% and a Nagelkerke *R* ^2^ of 0.496 (Table 2).

**TABLE 2:** Multivariable logistic regression predicting the diagnosis of peri‐implantitis.

Independent variable	Odds ratio (OR)	95% confidence interval	*p*
T allele of MMP‐8 −799C/T	3.93	1.23–12.55	0.020**
Upregulated MMP‐8 mRNA	6.57	2.25–19.18	0.001**
Elevated aMMP‐8 level (> 20 ng/mL)	7.49	2.85–19.69	< 0.001**

*Note:* Modelmetrics:
Classification accuracy: 81.4% Nagelkerke's *R* ^2^: 0.496
Binary logistic regression model including genetic (SNP), transcriptional (mRNA), and protein‐level (aMMP‐8) predictors.

** *p*‐values indicate statistically significant associations.

In the fully adjusted model, including smoking status and implant time in function—all three molecular predictors remained statistically significant: −799 T allele (OR = 6.04; *p* = 0.015), MMP‐8 mRNA upregulation (OR = 3.10; *p* = 0.039), and elevated aMMP‐8 levels (OR = 12.33; *p* < 0.001). Smoking (*p* = 0.369) and implant time in function (*p* = 0.997) were not significant covariates. The final model exhibited good fit (Hosmer–Lemeshow *p* = 0.917), a Nagelkerke *R* ^2^ of 0.408, and a classification accuracy of 73.8%.

## 4 Discussion

This study explored a non‐linear integration of genetic, transcriptional, and protein‐level features associated with peri‐implantitis. Of the three MMP‐8 SNPs investigated (−799C/T, −381A/G, +17C/G), only −799C/T showed allelic variation in this cohort. As a single SNP was evaluated inferentially, no correction for multiple comparisons was applied. Accordingly, all genetic findings should be interpreted cautiously, as they are exploratory in nature.

The −799 T allele was associated with peri‐implantitis but not with MMP‐8 mRNA levels. While MMP‐8 mRNA upregulation correlated with increased aMMP‐8 protein levels in PICF (OR = 4.44; *p* = 0.002), the T allele showed no transcriptional influence (OR = 1.50; *p* = 0.358). Nonetheless, all three variables—T allele (OR = 3.93; *p* = 0.020), mRNA upregulation (OR = 6.57; *p* = 0.001), and elevated aMMP‐8 (> 20 ng/mL; OR = 7.49; *p* < 0.001)—remained independent predictors in the final regression model.

The lack of association between this specific genotype and transcription suggests that parallel rather than sequential regulation is at play. Promoter SNPs like −799C/T may alter transcription factor binding without changing basal expression under stable conditions [5]. Epigenetic mechanisms (e.g., methylation, histone modification) can further influence gene accessibility in a context‐specific manner [6]. Post‐transcriptional controls, including microRNAs or RNA‐binding proteins, may modulate translation or stability of MMP‐8 transcripts [7]. Post‐translational modifications—such as glycosylation or proteolytic cleavage—can also affect the function of the MMP‐8 protein independently of transcription.

All participants had a prior history of periodontitis and were in a maintenance phase; thus, this variable lacked variation and was excluded from multivariable analysis. While this controlled for disease background, residual effects of subclinical inflammation cannot be excluded.

## 5 Conclusion

This study identified a combination of molecular biomarkers—namely, the presence of the MMP‐8‐ 799 T allele, upregulated MMP‐8 mRNA expression, and elevated aMMP‐8 protein levels—as independent predictors of peri‐implantitis within a well‐characterized cohort. While the multivariable logistic regression model demonstrated good discriminatory performance (AUC = 0.801; accuracy = 73.8%), these findings should be interpreted with caution. The model is exploratory and based on cross‐sectional, single‐center data without external validation. As such, it is not intended for immediate clinical application but rather to inform and guide future research on multi‐marker diagnostic strategies. Validation in larger, diverse, and prospective cohorts is essential before any clinical utility can be established.

## Disclosure

Permission to Reproduce Materials: No previously published material was reproduced in this article. All figures and tables are original.

## Ethics Statement

The study protocol was approved by the Ethics Committee of the School of Dentistry, Aristotle University of Thessaloniki (Approval Number: 115/25–05‐21).

## Consent

Written informed consent was obtained from all participants, including for the analysis of biological samples.

## Conflicts of Interest

The authors declare no conflicts of interest.

## Supporting information


**Data S1:** jre70026‐sup‐0001‐Supplementary Material.docx.

## Data Availability

The data that support the findings of this study are available from the corresponding author upon reasonable request.

## References

[1] S. Alassiri , P. Parnanen , N. Rathnayake , et al., “The Ability of Quantitative, Specific, and Sensitive Point‐Of‐Care/Chair‐Side Oral Fluid Immunotests for aMMP‐8 to Detect Periodontal and Peri‐Implant Diseases,” Disease Markers 2018 (2018): 1306396, 10.1155/2018/1306396. 30154936 PMC6098860

[2] A. Al‐Majid , S. Alassiri , N. Rathnayake , T. Tervahartiala , D. R. Gieselmann , and T. Sorsa , “Matrix Metalloproteinase‐8 as an Inflammatory and Prevention Biomarker in Periodontal and Peri‐Implant Diseases,” International Journal of Dentistry 2018 (2018): 7891323, 10.1155/2018/7891323. 30305812 PMC6165625

[3] T. Berglundh , G. Armitage , M. G. Araujo , et al., “Peri‐Implant Diseases and Conditions: Consensus Report of Workgroup 4 of the 2017 World Workshop on the Classification of Periodontal and Peri‐Implant Diseases and Conditions,” Journal of Clinical Periodontology 45 (2018): S286–S291, 10.1111/JCPE.12957. 29926491

[4] T. D. Schmittgen and K. J. Livak , “Analyzing Real‐Time PCR Data by the Comparative CT Method,” Nature Protocols 3, no. 6 (2008): 1101–1108, 10.1038/NPROT.2008.73. 18546601

[5] S. S. Nishizaki , N. Ng , S. Dong , et al., “Predicting the Effects of SNPs on Transcription Factor Binding Affinity,” Bioinformatics 36, no. 2 (2019): 364–372, 10.1093/BIOINFORMATICS/BTZ612. PMC7999143 31373606

[6] J. K. Choi and Y. J. Kim , “Epigenetic Regulation and the Variability of Gene Expression,” Nature Genetics 40, no. 2 (2008): 141–147, 10.1038/NG.2007.58. 18227874

[7] P. Ivanov and P. Anderson , “Post‐Transcriptional Regulatory Networks in Immunity,” Immunological Reviews 253, no. 1 (2013): 253–272, 10.1111/IMR.12051. 23550651 PMC6989036

