# Supplementary material for: MMP‐8 in Peri‐Implantitis: A Cross‐Sectional Study on Genetic Polymorphisms and Enzymatic Activation
Source: J Periodontal Res. 2025 Aug 7;61(1):95–7. doi: 10.1111/jre.70026 (PMC12961359; doi:10.1111/jre.70026)
Supplement: Supplementary file 1 — Data S1: jre70026‐sup‐0001‐Supplementary Material.docx. [file JRE-61-95-s001.docx]

**Supplementary Material**

**Suppl Information on Materials and Methods**

**Detailed Sampling Timeline**

Participants underwent an initial clinical and radiographic examination to classify peri-implant tissue status. Biological sample collection was scheduled one week after clinical assessment to avoid probing-induced biomarker alteration. Sampling was performed between 8:00 and 10:00 AM to minimize diurnal variation. Participants refrained from eating, drinking, smoking, and performing oral hygiene procedures for at least 8 hours before sample collection.

**Inclusion and Exclusion Criteria**

Eligible participants were adults aged 18 years or older, with at least one functional dental implant in place for over 12 months. Exclusion criteria included uncontrolled systemic diseases (e.g., diabetes mellitus), pregnancy, lactation, recent (within 3 months) use of systemic antibiotics or anti-inflammatory drugs, recent periodontal or peri-implant therapy (within 6 months), and current smoking or recent smoking cessation (<12 months). Only one implant per participant was included to maintain statistical independence.

**Sample Size Calculation**

The sample size was calculated separately for genetic and biomarker analyses.

For genetic analysis, assuming an expected minor allele frequency of 40% and an odds ratio of approximately 2.0 for peri-implantitis susceptibility (Emingil et al. 2014), a minimum of 45 participants per group was needed for 80% power at α = 0.05.

For aMMP-8 level comparison, pilot data suggested a Cohen’s d of 0.71, requiring at least 30 participants per group for 80% power.

Thus, the final sample size of 124 exceeded minimum requirements, ensuring adequate statistical power across all analyses.

**RNA Extraction and cDNA Synthesis Protocol**

Peri-implant crevicular fluid (PICF) samples were stored at −80°C prior to RNA extraction. Total RNA was extracted using the Quick RNA Mini Prep Plus Kit® (Zymo Research, USA), following the manufacturer’s protocol. RNA purity and concentration were evaluated using a NanoDrop™ spectrophotometer. Reverse transcription to complementary DNA (cDNA) was performed using the GoScript™ Reverse Transcription System (Promega, USA), applying both oligo(dT) and random primers.

**PCR Amplification and Sanger Sequencing Details**

Genomic DNA was extracted from unstimulated saliva samples using the Quick-DNA Miniprep Plus Kit® (Zymo Research, USA). PCR amplification targeted the −799C/T (rs11225395), −381A/G (rs1320632), and +17C/G (rs2155052) polymorphisms of the MMP8 gene. Specific primers were used, and thermocycling conditions were optimized. PCR products were visualized by 2% agarose gel electrophoresis and sequenced using Sanger sequencing (PlateSeqSupreme®, Eurofins Genomics Europe).

**aMMP-8 Quantification Method**

Active MMP-8 (aMMP-8) levels in peri-implant crevicular fluid (PICF) were quantified using the ImplantSafe® lateral-flow immunoassay in conjunction with the ORALyzer® digital reader (Dentognostics GmbH, Jena, Germany). The diagnostic threshold of **20.0 ng/mL** was selected based on external validation studies that demonstrated this cutoff's ability to reliably differentiate peri-implantitis from healthy or mucositis-affected sites.

In particular, Sorsa et al. (2017) and Lähteenmäki et al. (2022) reported that a threshold of 20 ng/mL provided optimal sensitivity and specificity for clinical diagnosis using the same point-of-care platform. Additionally, Xanthopoulou et al. (2024) confirmed the robustness of this cutoff in a Greek population using the ImplantSafe® system, further supporting its use in the present study. Based on these findings, we adopted this clinically validated value to ensure comparability with previous literature and facilitate clinical translation of our results (Lähteenmäki et al. 2020; Sorsa et al. 2017; Xanthopoulou et al. 2024).

Κάντε κλικ ή πατήστε εδώ για να εισαγάγετε κείμενο.

**Randomization and Blinding Procedures**

Biological samples were assigned anonymized numerical identifiers at the time of collection. The analyses of RNA, DNA, and aMMP-8 levels were conducted blind to the participants' clinical diagnosis. Sample processing batches were randomized to reduce inter-assay variability.

**Statistical Analysis Details**

Statistical analyses were conducted using IBM SPSS v27.0. Nonparametric tests (Mann–Whitney U, chi-square) were used for group comparisons. Stepwise binary logistic regression was employed for multivariable modeling, with entry at p < 0.05 and removal at p > 0.10. A receiver operating characteristic (ROC) analysis was performed to evaluate the biomarker. Missing data (<5%) were handled via complete case analysis.

________________________________________

*Supplementary Figures*

### *Figure S1: ROC Curve Analysis for aMMP-8 Levels in Predicting Peri-Implantitis*

| *Cut-off (ng/mL)* | *Sensitivity (%)* | *Specificity (%)* | *Area Under the Curve (AUC)* | *p-value* |
| --- | --- | --- | --- | --- |
| *20.0* | *76.5* | *70.4* | *0.801* | *<0.001 *** |

*
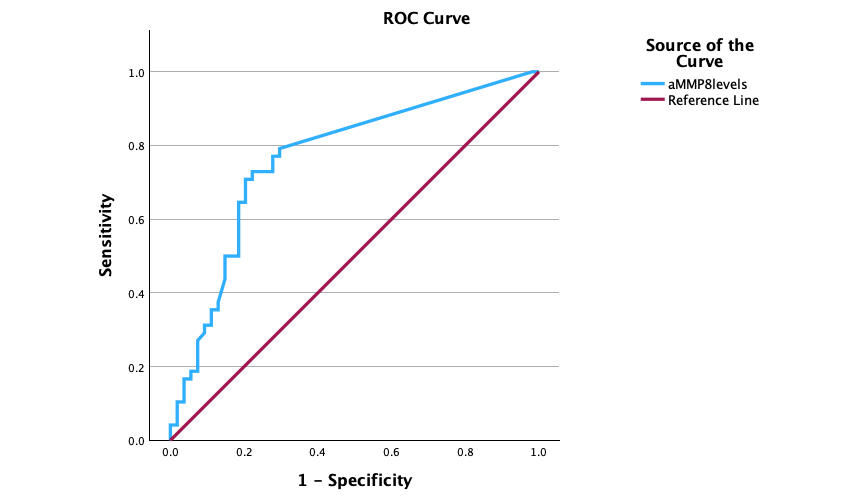
*

*AUC value (0.801) indicates good diagnostic accuracy of aMMP-8 for peri-implantitis.*

*Analysis was performed using binary disease outcome as a dependent variable and aMMP-8 concentration as a continuous predictor.*

**Exploratory Analysis of the Genotype–Transcript–Protein Cascade**

To explore the potential biological pathway linking MMP-8 −799C/T gene polymorphism, mRNA expression, and active MMP-8 (aMMP-8) protein levels in peri-implantitis, we conducted a **serial binary logistic regression analysis**. Specifically, we assessed the sequential associations among: (1) the presence of the T allele at the −799C/T SNP; (2) upregulated MMP-8 mRNA expression (defined as fold change >2.0); (3) elevated aMMP-8 protein levels in PICF (defined as >20.0 ng/mL); and (4) diagnosis of peri-implantitis.

The results were as follows:

- **T allele and MMP-8 mRNA**: No significant association was observed between the −799T allele and mRNA upregulation (OR = 1.50, 95% CI: 0.63–3.56, *p* = 0.358);
- **mRNA and aMMP-8**: Upregulated MMP-8 mRNA expression was significantly associated with elevated aMMP-8 levels (OR = 4.44, 95% CI: 1.74–11.32, *p* = 0.002);
- **Final multivariable model**: All three predictors — T allele presence, mRNA upregulation, and elevated aMMP-8 — were independently associated with peri-implantitis in the final logistic regression model (T allele: OR = 3.93, *p* = 0.020; mRNA: OR = 6.57, *p* = 0.001; aMMP-8: OR = 7.49, *p* < 0.001), with an overall classification accuracy of 81.4% and Nagelkerke R² = 0.496.

Taken together, these findings suggest that **MMP-8 mRNA expression does not mediate the genetic effect of the −799T allele** on protein-level aMMP-8 expression or disease presence. This supports the hypothesis that **alternative regulatory mechanisms**—including post-transcriptional (e.g., microRNA interference, mRNA stability), translational, or epigenetic processes—may modulate the observed associations. While a formal mediation analysis was not performed due to statistical power constraints and cross-sectional design, this exploratory model informs future longitudinal and mechanistic studies.

Emingil, Gülnur, Buket Han, Ali Gürkan, Afig Berdeli, Taina Tervahartiala, Tuula Salo, Pirkko J. Pussinen, Timur Köse, Gül Atilla, and Timo Sorsa. 2014. “Matrix Metalloproteinase (MMP)‐8 and Tissue Inhibitor of MMP‐1 (TIMP‐1) Gene Polymorphisms in Generalized Aggressive Periodontitis: Gingival Crevicular Fluid MMP‐8 and TIMP‐1 Levels and Outcome of Periodontal Therapy.” *Journal of Periodontology* 85(8):1070–80. doi:10.1902/jop.2013.130365.

Lähteenmäki, Hanna, Kehinde A. Umeizudike, Anna Maria Heikkinen, Ismo T. Räisänen, Nilminie Rathnayake, Gunnar Johannsen, Taina Tervahartiala, Solomon O. Nwhator, and Timo Sorsa. 2020. “Ammp-8 Point-of-Care/Chairside Oral Fluid Technology as a Rapid, Non-Invasive Tool for Periodontitis and Peri-Implantitis Screening in a Medical Care Setting.” *Diagnostics* 10(8). doi:10.3390/diagnostics10080562.

Sorsa, Timo, Dirk Gieselmann, Nicole B. Arweiler, and Marcela Hernández. 2017. “A Quantitative Point-of-Care Test for Periodontal and Dental Peri-Implant Diseases.” *Nature Reviews Disease Primers* 3(1).

Xanthopoulou, Vithleem, Ismo T. Räisänen, Timo Sorsa, Dimitrios Tortopidis, and Dimitra Sakellari. 2024. “Diagnostic Value of AMMP-8 and Azurocidin in Peri-Implant Sulcular Fluid as Biomarkers of Peri-Implant Health or Disease.” *Clinical and Experimental Dental Research* 10(3). doi:10.1002/cre2.883.
